# Improving childhood nutrition and wellness in South Africa: involving mothers/caregivers of malnourished or HIV positive children and health care workers as co-designers to enhance a local quality improvement intervention

**DOI:** 10.1186/s12913-016-1574-4

**Published:** 2016-08-05

**Authors:** Claire van Deventer, Glenn Robert, Anne Wright

**Affiliations:** 1 Department of Family Medicine and Rural Health, University of the Witwatersrand, Phillip Tobias Building, York Road, Parktown, Johannesburg, South Africa; 2 Health Care Quality & Innovation, Florence Nightingale Faculty of Nursing & Midwifery, King’s College London, James Clerk Maxwell Building, 57 Waterloo Road, London, SE1 8WA UK; 3 University of the Witwatersrand, Phillip Tobias Building, York Road, Parktown, Johannesburg, South Africa

**Keywords:** Experience-based Co-design, Chronically ill children, Quality improvement, Child nutrition, Co-design, HIV

## Abstract

**Background:**

A significant proportion of children admitted to a hospital in a South African sub-district in 2010 were severely malnourished and - when concurrently HIV positive - were not correctly initiated on antiretroviral therapy. Audit data over a subsequent four year period revealed that 60 % of malnourished children admitted to the hospital were HIV positive. To supplement an ongoing local quality improvement (QI) intervention addressing poor nutritional outcomes in children in this setting, Experience-based Co-design (EBCD) was used to enhance previously low levels of mother, carer and staff engagement.

**Methods:**

EBCD was implemented over an 8 month period. Non-participant observation was conducted comprising a total of 10 h in 5 different clinical locations. Semi-structured interviews were undertaken with 14 purposively selected staff members as well as 10 mothers/caregivers. The staff interviews were audio-taped whilst the mothers/caregiver interviews were filmed; both sets of experiences were analysed for key ‘touchpoints’. Mothers/caregivers and staff participated in separate feedback events and then came together to identify their shared priorities for improving the service. Participants worked together in 3 smaller co-design teams to implement improvements.

**Results:**

There was overlap in staff and mother/carer views as to their priorities for QI. However, whilst staff typically highlighted pragmatic issues, mothers/caregivers were more likely to identify experiential and relational issues. A total of 38 QI interventions were proposed after the priorities had been discussed and delegated to the 3 co-design teams; 25 of these changes had been implemented or were being planned for by the end of the study period. Examples included: a point of care blood machine being bought to shorten the time in the emergency department whilst waiting for laboratory results; a play area being organised for children attending the HIV clinic; the development of three standard operating procedures to improve clinical handover and waiting times; and privacy screens installed to improve privacy in reception.

**Conclusions:**

The impact of EBCD was noted both in practical improvements focused on a better experience for mothers/caregivers and children within the system and in reflections from stakeholders as to the value added to the ongoing QI intervention by the co-design process.

**Electronic supplementary material:**

The online version of this article (doi:10.1186/s12913-016-1574-4) contains supplementary material, which is available to authorized users.

## Background

Concerns about the wellbeing of children have increased over the last few decades and these are reflected in United Nations Children’s Fund (UNICEF) [1] and World Health Organisation (WHO) [2] guidelines as well as the Millenium Development Goals (MDGs) [3] where Goal 4 was specific to the reduction of child mortality. The newest global strategic document, Sustainable Development Goals (SDGs) has also captured the importance of child health and nutrition in Goal 3 (Good health and wellbeing) [4]. A large contributor towards child morbidity and mortality, especially in developing countries, is malnutrition. Coupled with Human Immunodeficiency Virus/Acquired Immunosuppressive Disease Syndrome (HIV/AIDS), this has become a crippling burden to these populations.[5] South Africa has been unable to attain MDG goal4 set for 2015, regarding child health indicators [6].

Routine health data in a district in the North West province, South Africa, indicated that it was performing relatively poorly in 2010 in relation to severe malnutrition admissions for children [3]. The district comprises four sub-districts with the research subdistrict containing 9 primary health care (PHC) clinics (plus two mobile clinics for farms and outlying areas), all of which refer patients to a secondary hospital in this subdistrict. The hospital is in a large university town comprised of suburbs and surrounding townships. Most of the children admitted to the hospital come from an adjoining township which comprises formal (bricks and cement) and informal housing (tin shacks). In the hospital there is a general paediatric ward and a general paediatric outpatient clinic (OPD). In addition to the general outpatient clinic, there is a special Human Immunodeficiency Virus (HIV) clinic serving adults and children who are HIV positive. All the PHC clinics also manage malnourished and HIV positive children.

In the period October to December 2010, 14 % of children under 5 years old admitted to the hospital were severely malnourished. In the district as a whole 6 per 1000 children were diagnosed with severe malnutrition (compared to a national average of 4.8 per 1000 children [3]), placing the district in the bottom quartile of all districts in South Africa. Additionally only 50 % of HIV positive under one year olds admitted to the hospital were initiated on Antiretroviral therapy (ART), contravening National ART guidelines [7].

In response, a multi-disciplinary quality improvement (QI) project involving a ‘QI task team’ was initiated in 2011 by the district family physician who is a primary care specialist concerned with clinical governance and patient centred care.. To begin, a range of audits were conducted to provide a baseline relating to various points in the service where children were seen; for example, the paediatric ward, the children’s HIV clinic and the PHCs within the sub-district. The audits involved collating and analysing information from hospital files and handheld child booklets; nutrition students from a local university also examined breastfeeding practices and costed the different milk alternatives available to mothers/caregivers.

**Fig. 1:**
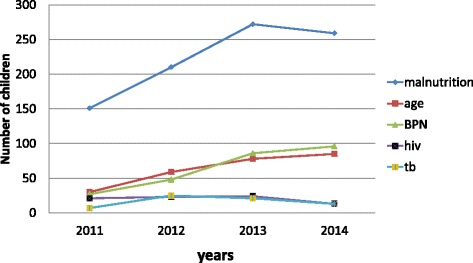
Trends in the most common admission diagnoses associated with malnutrition (2011–14)

One ongoing audit - based on the admission register of the paediatric ward - was designed to monitor any changing patterns of malnutrition in the period from the start of the QI intervention in 2011 until June 2015. Common reasons for admission were bronchopneumonia (BPN), gastroenteritis (GE) and HIV related illnesses. There was however a large number of children admitted for malnutrition only (855 out of 1511 children over this period). For example, in 2011 60 % of the children in the audit were admitted with a sole diagnosis of malnutrition although this had decreased by 2014 to 45 % with the remainder of the admitted children having co-morbidities. Although BPN, GE and malnutrition alone had increased over the 4 year period as reasons for admission, HIV and TB had slightly decreased (Fig. 1).

A further concern raised by this audit was that in this period, 30 % of children who had been admitted primarily for a surgical procedure were found at the admission audit to have concurrent malnutrition and in 50 % of these cases the diagnosis of malnutrition was not made and managed by the clinical team but only identified retrospectively from the ward register by the researcher conducting the audit.

The audit of the ward register also revealed that from 2011 to 2014 60 % of all malnourished children admitted had HIV/AIDS (although not all were admitted for reasons relating to this). There has been a significant decrease in the numbers of children diagnosed with HIV in South Africa over recent years - reflecting the nation-wide success of the Eradication of Mother to Child Transmission (EMTCT) programme [8]. The following illustrates the diminishing prevalence of HIV in the lower age groups as a practical wxample; of the 105 children still being seen at the HIV clinic at the study hospital in January 2015, 49 were in the 10–14 year old group, 25 were in the 5–9 year old group, 27 comprised the 1–4 year old group and only 4 were under one. It was perhaps initially surprising to find that there was a high level of malnutrition (stunting in particular) in the older groups of children with HIV, up to the age of 14 years with 67.3 % of them having inadequate growth. However, older children are often not cared for by their mothers (most of whom have died) and are known to frequently default their medication. Blood results at the hospital indicate that more than half of these children (of all ages) are not virologically suppressed (although they have good CD4 counts). Unsatisfactory levels of suppressed viral loads were found for all except the under one year olds, who seem to have benefited the most from PMTCT.. This small cohort of children have remained at the hospital HIV clinic and not been down referred to PHC clinics, due to either complications or poor patterns of adherence and are thus the most vulnerable group.

**Fig. 2:**
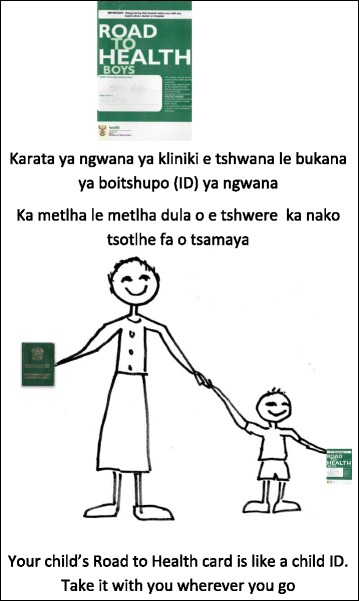
Poster encouraging parents to carry their child’s health booklet with them

A majority (57.5 %) of the malnourished children were from a relatively small area in an adjoining township; knowing this was useful in targeting, firstly, support to the relevant PHC clinics and, secondly, community information sharing. The work of the QI task team from 2011 included health information material being created and distributed widely to PHCs and incorporated the concept of a child identity document (the ‘Road-to-Health’ card) which was intended to accompany parents wherever they went (see Fig. 2).

A hospital-community discharge link was created enabling patients to be identified and followed at the PHCs by dieticians; this new pathway included support for patients with identity documents and food parcels. A patient tracing system was also initiated. On the paediatric ward, HIV counsellors joined ward rounds to ensure that all HIV positive children were initiated on treatment as soon as possible; this led very rapidly to 100 % of HIV positive children being started on ART.

However, ongoing audit results showed that 881 children whose weight was unsatisfactory had been admitted between 2011 (when the QI intervention began) and 2014; the year-on-year results showed that 14.4 % of all admitted children in 2011 were malnourished, in 2012 this figure was 11.6 %, in 2013 16.1 % and in 2014 15.1 % suggesting no overall improvement in malnutrition rates during the first 4 years of the QI intervention. And so in spite of the efforts described above, there seemed to be no decrease in admissions of malnourished children to the paediatric ward. There was also no decrease in readmissions of children with malnutrition (7 readmissions in 2011; 4 in 2012; 9 in 2013 and 9 in 2014). On reviewing these results in late 2014 there was a concern by the QI team that parents of children had never been directly involved in the QI work; it was felt that they might be a useful additional resource in improving childhood malnutrition rates in the sub-district.

There is an increasing evidence base that greater patient engagement leads to improved outcomes [9–13]. A systematic review of the evidence by the author found:

‘’There are barriers to patients’ participation in QI in health and in spite of policy support for user involvement in QI, it is a difficult strategy to implement … there are enablers to patients’ involvement in QI: when patients are involved in QI efforts in health care, there are innovative, often unexpected outcomes at different levels of the process and sustaining these efforts is possible with ongoing individual or group support” [14].

One of the most authentic approaches to client engagement appeared, based on the systematic review, to be Experience-based Co-design (EBCD) where quality improvements are co-created - and jointly implemented - by staff and their patients [15–20]. The EBCD approach - based on a combination of Participatory Action Research, user centred design, narrative approaches and learning theory [21] - explicitly recognises that the experience and feelings of patients regarding their interactions with health care services and staff is an important - but often neglected - component of health care quality. For example, a letter written in the local newspaper in the research sub-district by a mother in June 2015 reads (translated from Afrikaans) as follows:

‘’I went to the emergency department (with a sick 5 year old) … I was chased out by a female doctor. I went to open a file and the person who helped me was also not the friendliest, didn’t greet me, was just plain rude … The doctor told my child to vomit in a medical waste holder. There was no silver container for him. While the child waited for test results he started vomiting again and one (nurse) shouted to the doctor, ‘doctor the child is throwing up again’. The doctor simply said:’There is nothing wrong with him, give him the medicine’. They just sat there while my child had to throw up in a dustbin” [22].

This child survived and was not at risk at any time. Nonetheless, according to the mother this was, unsurprisingly, ‘’ a very traumatic experience for us”, succinctly illustrating the impact of the lack of an aesthetic element in healthcare interactions that needs to be recognised and incorporated into QI activities.

**Fig. 3:**
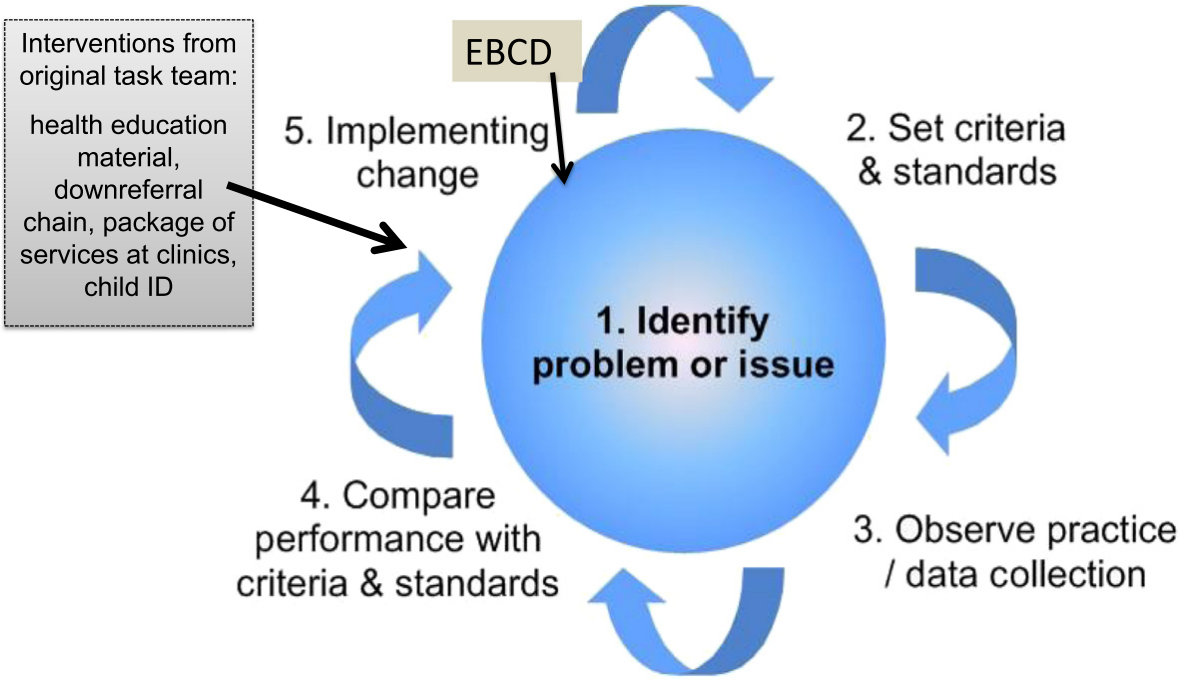
The inclusion of EBCD in the ongoing QI intervention

Whilst it is acknowledged that - in the short term - EBCD largely brings about incremental QI changes rather than transformational or dramatic improvements, ‘’ … the individual and collaborative work underpinning these small changes lies at the root of deeper changes in attitudes and behaviours as well as other valuable legacies from EBCD projects” [13]. The approach has been used in healthcare QI work since 2005 and more than 50 projects have been identified internationally [20–25]. Having read extensively about patient involvement in healthcare and in QI in particular and having completed a systematic review on this topic, EBCD therefore appeared to the researcher to be an appropriate method for increasing the involvement of mothers/caregivers in the ongoing QI work and so the approach began to be implemented in January 2015 alongside the ongoing local QI intervention (Fig. 3). For the purposes of clarity, the combination mothers/caregivers will be used throughout as children are frequently cared for by other family members due to the death of the mother or for other social reasons.

As illustrated above, a quality cycle usually includes the identification of a problem; the setting of standards; collection of baseline data; a comparison of actual practice with these standards and then planning and implementing change in order to meet the standards that were set. The original QIP had utilised this model and interventions have been briefly discussed above. EBCD was seen as a second round of interventions to add value to the first cycle.

The aim therefore of incorporating EBCD into the QI intervention in this way was to enhance childhood nutrition and wellness in the sub-district by increasing patient inclusivity in the ongoing improvement work which had begun in 2011. The objectives towards this aim included exploring the:

- understanding and perceptions of mothers/caregivers of their experiences of health care services for their children;
- providers’ experiences with the care they provide towards mothers/caregivers with children with malnutrition and/or HIV;
- the collaborative planning and implementation of solutions by mothers/caregivers and staff.

The methods to achieve these objectives are described in detail in the following section.

## Methods

The implementation of the EBCD approach was undertaken with a group of mothers/caregivers from the paediatric ward and the child HIV/wellness clinic sited in the most vulnerable geographic areas as identified in the audit data, as well as with staff from all of the relevant service provision areas. There were hospital matrons (2) and professional nurses (3), medical officers (2), paediatricians (2), dieticians (2), a social worker, an HIV counsellor and a clinical associate involved and the project leader was a family physician. Most of these personnel members had been part of the original QI. However, in terms strategic involvement, other roleplayers eg the screening nurse at the reception area, were purposively included as well. A core team of 4 health professionals co-ordinated and facilitated all stages of the EBCD process including the feedback and co-design meetings. The core team comprised a nutritionist, a paediatric nurse, a family physician and a paediatric doctor. Regular bi-monthly written reports were submitted to the hospital management on the progress of the project.

Mothers/caregivers and staff members were invited to participate in the EBCD process through purposive sampling. Participating staff included a range of professionals involved with caring for the children, as described in the previous paragraph. Mothers/caregivers were recruited through both the HIV wellness clinic and the paediatric ward, both found within the previously described secondary hospital. The inclusion criteria were all mothers/caregivers with a child either (a) having being diagnosed with malnutrition and/or any other relevant condition - for example, gastroenteritis, pneumonia or HIV - from the township or (b) with children from the same area who were HIV positive and were being managed at the HIV clinic. All participants had to be able communicate in English or Afrikaans as the researcher is fluent in both. Written consent was obtained from all participants.

### Process of EBCD [16]

The core project team oversaw the EBCD process after formal agreement was granted from the hospital management. Ethics approval for the study had also been granted from the University of the Witwatersrand and the provincial research committee. Written signed consent was obtained from each participant, (mothers/caregivers and staff members) after an information letter was read to each one of them explaining the project. Non-participant observations were undertaken by the researcher along the patient journey through the health care system and included 5 key service areas where the patients come into contact with the health care system in order to provide initial insights into the subtleties of patient experiences [26, 27]. The areas chosen for observation were the reception area, two rooms within the children’s ward, the HIV clinic on the paediatric outpatient day and the general paediatric clinic. Field notes were taken using a template from the EBCD workbook (Additional file 1) [28] and kept as narrative evidence.

The next step was to interview staff (14) and gain an understanding of their perception of the service in which they worked and their views on how it could be improved. The staff cohort purposively chosen for interviews encompassed a range of professionals involved with caring for the children: four doctors (including medical officers and paediatricians), two paediatric nurses, two dieticians, one social worker, a clinical associate, a counsellor, two matrons and a triage nurse at reception. The interviews were audio-taped. They were then analysed by transcribing the information and colour coding the different themes (7) which emerged. This was fed back to a meeting with staff where facilitated discussion about all the issues led to a set of priorities for improvement being identified and documented.

Concurrently, videotaped semi-structured interviews were conducted with mothers/caregivers. According to ethical prescripts from the University Ethics committee, all these tapes are being kept in a secure environment for 5 years. The mothers/caregivers responded to open-ended questions relating to their experiences within the health care system. Questions were asked regarding the mother’s experience at each contact point in her journey as well as her ideas in relation to possible improvements that should be made.

The interview concentrated on ideas and emotions that were elicited and not on a set of rigid questions. Each mother/caregiver was individually shown the sections of her own interview that had been edited into a shortened compilation film. This film was structured around the common themes which had emerged in the interviews. This preview of the film by each mother/caregiver was in order both to validate what was to be shared and to allow each woman the choice to remove any material with which she felt uncomfortable.

**Fig. 4:**
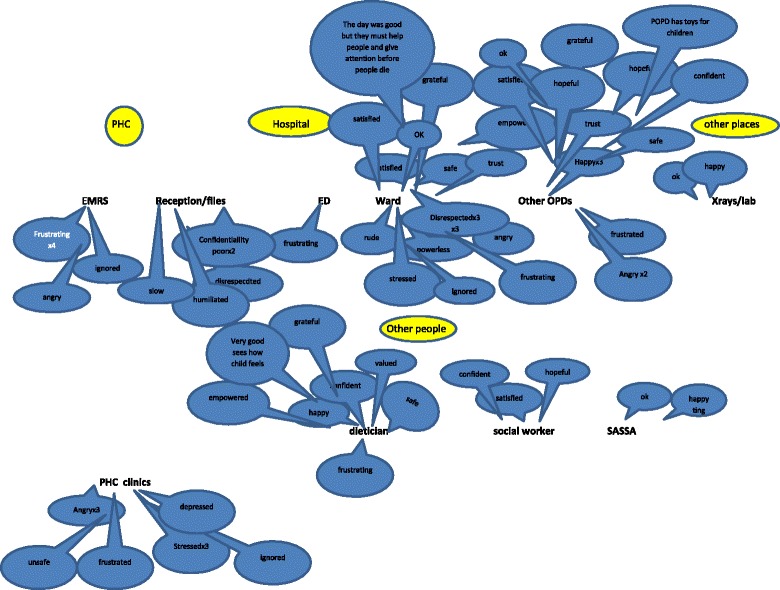
Emotional mapping done with mothers of chronically ill children

**Fig. 5:**
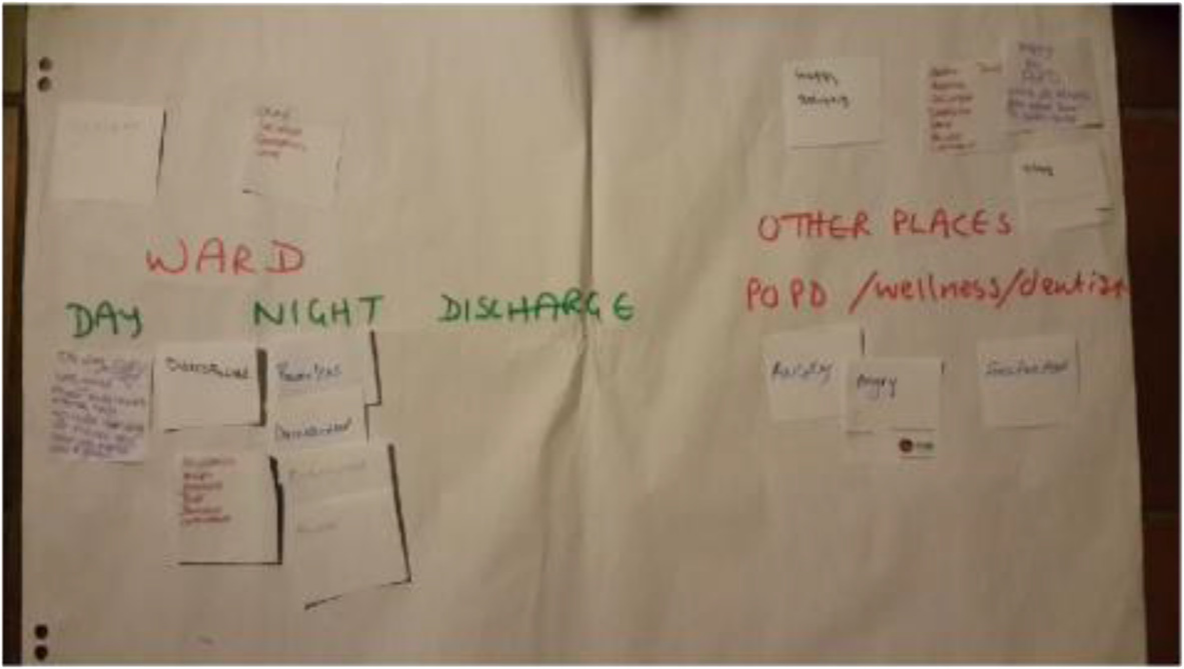
Part of the emotional mapping pathway done by mothers

The mothers/caregivers were then invited to a feedback meeting where the film was shown and attendees shared their positive and negative experiences. A facilitated emotional mapping exercise [29, 30] (see Figs. 4 and 5 in results section below) was used as part of the reflective group process to enable the participants to develop their own set of improvement priorities. Emotional mapping involved the mothers/caregivers applying emotion words (positive or negative) to the key points in their experience of interacting with any part of the health care system.

Twenty five mothers or caregivers were contacted and invited to be part of the research. Two declined outright and thirteen others did not respond to the request. However the 10 mothers/caregivers who agreed to be interviewed were willing to be transparent about both strengths and weaknesses in the health care system as they perceived them. All of the mothers/caregivers except one were relatively fluent in either English or Afrikaans. A video recording was made of each mother during the open ended interview format. One interview was excluded due to the poor quality of communication, resulting in 9 videotaped interviews. These videos were then watched a number of times by the researcher (CvD) and key themes from each recording identified. Clips from each video illustrating the themes were then tagged and organising according to each theme. The total video time of 208 min was reduced to 40 min and presented as a short film which could be played back to patients and staff.

A combined staff-patient event was the forum where the emerging improvement priorities from the staff and patient feedback events described above were shared and 3 mixed patient-staff co-design groups were then formed to work on specific improvement priorities as part of the QI process. These groups then sought to identify and implement practical solutions for change over a period of two and a half months.

Finally, a ‘celebration’ event was held to which all staff and mothers/caregivers were invited to showcase the changes and assess what further work needed to be done.

## Results

### Observational fieldwork

The pathway a mother with an ill child entering the hospital was expected to take, was observed at five points (total observation time was 10 h across the 5 areas) and the findings were incorporated into the report that was fed back at the staff meeting (see below). A number of disturbing practices were noted as well as other very good processes and high quality care.

For example, in the reception area a mother was informed by the screening nurse - with a patronising and arrogant attitude - that the fact that her child had a fever had nothing to do with the child’s epilepsy medication (the mother’s illness narrative) but rather because the child was overdressed. In the same observation period, an adult patient was asked why she had come to hospital and - with twenty or more fellow patients watching - said she had a chest pain and started pulling up her shirt to indicate the area. She was immediately stopped and severely reprimanded by the same nurse, to the general astonishment of everyone in the waiting room. A common complaint in the patient interviews (see below) was that of extended waiting times and this was confirmed during an observation at the HIV clinic where patients waited for many hours for a doctor to arrive. Children were irritable and running around in the clinic, as there was no activity area available for them.

In contrast to these negative experiences, in the children’s ward a nurse entering the 6-bedded ward early in the morning made a point of introducing himself to each mother individually, greeted them with a handshake and cheerfully asked about their children. On this same ward, there were bright pictures on the walls and all the staff members gave attention to the mothers, including the cleaners and clerks. However, two safety issues were noted: a baby was observed crawling in the corridor whilst the polisher with a long electric cord was being used on the floors and a lumbar puncture on a child - who had been admitted the previous night in a stuporose condition - had not yet been performed due to poor handover procedures.

### Staff interviews and feedback event

The 14 consenting professionals participated in open-ended, audio taped interviews lasting between 20 and 60 min. Analysis of the staff interviews was reflected back at the staff meeting and focused on 7 themes:

- emotion
- logistics (waiting; triage; space; clinical protocols)
- patient factors
- staff attitudes
- communication
- teamwork
- continuity of care

An unanticipated theme was that of ‘emotion’; it was evident that all the interviewees had been significantly affected at some point by either anger, sorrow, guilt, memories of patients, enthusiasm etc. Many of these responses were very self-reflective and sometimes painfully honest. The themes are summarised in Table 1 with illustrative excerpts from the interviews for each.

**Table 1:** Staff themes

Theme	Illustrative quotations
Emotion	‘’To work with children is wonderful… you can play and pull your face. It is so satisfying” ‘’ I have an instinct for this” ‘’ We get emotional because we care”, ‘’ I have an emotional connection”. ‘’I am passionate..” “I lost it…” ‘’I get so angry..” ‘’I have to say this thing (malnutrition) kills.. It is heart wrenching”, ‘’It makes me so sad”
Logistics	‘’It takes hours waiting for files .. It is ridiculous .. They are so tired when they get to us.” ‘’Unnecessary referrals from PHC” ‘’ Patients know what to tell us..(referred from ED to clinic).. “’I am not stupid, I will come back after 4”’
(a) waiting	
(b) triage	‘’Triage is done by students with no insight.” ‘’Get rid of triaging and go back to ‘first come first served’
(c) space	‘’The new POPD is very nice” ‘’ We need a sound proof room for counselling.” ‘’Mothers sleep on the floor”. ‘’Move to the ground floor so that we can have a play area outside”
(d) clinical protocols	‘’The 10 steps has helped such a lot”, ‘’ we do in-service every day”
Patient factors	‘’There are changing caregivers all the time with lack of responsibility”. ‘’It seems the children blame themselves especially where the mothers passed away..this sometimes leads to overdose” ‘’They really don't know..80 % don't know babies can eat protein” ‘’Rooibos (tea). It comes from the grannies ..They see it as medicine” ‘’Mothers are hungry for information . They want to do the right thing and be better parents” ‘’Parents are passive” ‘’The mothers hide this (that they have given muti/traditional medicine)” ‘’Some of the grants go for alcohol” ‘’Some lie…” ‘’Sometimes she cannot read so she misses the appointment” ‘’Their hair is done, they are wearing nice clothes. You don't see suffering on the side of mothers..how did it come to this?” ‘’It is their culture..The ‘raad’ ‘’(traditional medicine)
Attitude	‘’I feel so bad when I have that heart and others don't” ‘’Certain patients are easier targets” ‘’I have seen mothers in tears who don't want to talk....she was told she isn't a good mother” ‘’Patients feel judged” ‘’The dietician confronted the nurses..They were so mean to the mother after that” ‘’Guilt and blame is projected by us” ‘’Sister is sometimes impatient..I tell them it is not the patient’s fault” ‘’We should move away from the blame and listen to the reasons why certain things happen” ‘’I don't shout at them..I try and compose myself” ‘’Our nurses are respectful” ‘’I will never greet you from a distance” ‘Some mothers need to be "shouted" ‘’Some people in the ward create a good culture..It makes a difference” ‘’The staff at ward.X …are very warm, very kind, very welcoming. That kind of atmosphere.. There is a culture”
communication	‘’The mother gets some relief where someone is listening” ‘’If you remain calm and explain they become receptive and say they will try. If you attack them they shut off..they don't even look at you when you come in the room” ‘’Encourage them by showing them increases in weight” ‘’It was said by the nurses that the mother was in tears because she didn't have an ID and no one had asked” ‘’I try to find out the reasons” ‘’Sometimes the mother was alone, there was no food, the father had left and she tried to get money..when they tell you their story their challenges…” ‘’After the news was broken that was the difficult moment” ‘’I had to learn the language, this really made a difference” ‘’I play with the baby if I cannot open the door to the parents..that is my door” ‘’I sometimes tease the mothers who sleep on the floor just to get the spirits up” ‘’The new doctors don't understand the struggle and say..just come back next week without thinking, then they get angry when the mother doesn't come back” ‘’Most of them..It is the wrong message the media is giving” ‘’I try and give a lecture. Some are thankful and others resistant” ‘’I explain that (related to traditional medicines) anything that goes into the mouth or rectum is dangerous and it is all about dosage. Pour Harmanns (folk medicine) into water and wash, don't drink, to protect against spirits” ‘’We were discussing a child and they (the doctors) started laughing. The mother started crying because she thought we were laughing at her child. The interns don't always realise the parents are listening” ‘’It is difficult to carry the message across”
Team	‘’It's important that the social worker sees them-she gets the true background and the mother gets some relief” ‘’There is great team work” ‘’Lots of support and interest from my manager” ‘’The safety net (of the clinic nurses) is not so strong. There is a lack of clinical sharpness/attention to detail” ‘’Feedback from team members is not always good” ‘’With each and every visit they must go to the counsellor and social worker.” ‘’When you call a doctor 4–5 times and he doesn’t pick up…those who get into trouble are the nurses” ‘’The staff is like minded. The dietician, doctors and nurses-children are important for them” ‘’The front clerks and I sometimes fight” ‘’We are working in a team and if the chain breaks…”
Continuity	‘’The paediatricians are very focussed and there is some continuity” ‘’Some of them have been there for years and years” ‘’She (HIV positive young woman) has a baby who is negative. I have known her for 10 years” ‘’They know the children and recognise the story is going on. This affects the way they are managed. I have already treated this child. This is the third time … this is now serious”

At the staff feedback meeting where 24 members attended, 9 areas for improvement were identified. These were practical interventions that could be dealt with immediately, such as buying mobile screens for the reception area in order to increase privacy and budgeting for a point of care blood machine to reduce laboratory waiting time for sick children in the emergency department.

### Mother and caregiver interviews and feedback event

Overall, the mothers/caregivers’ stories contained positive and negative elements. A common positive phrase in respect of the OPD, HIV clinic and ward was ‘they took me good’, meaning they ‘treated me well’. A number of individual staff were cited by name as being particularly helpful or supportive. For example, one doctor was praised for calling a child ‘her chocolate baby’ and giving the child a chocolate every time they visited her; another staff member for encouraging a mother that her child would get better. Whilst 3 of the mothers - despite the rough conditions - found the inpatient experience tolerable, other mothers referred to it as hell, and the staff as ‘rude, really rude’. A number of mothers said they were persevering with the service for the lives of their children and seemed not to mind the sacrifice of their personal discomfort. Other mothers said health workers should leave their personal issues at home as it seemed as if they brought them to work with them. There were also allegations of rough handling of children and a lack of assistance with feeding of younger, helpless patients. It was clear that some mothers/caregivers expected much more from the service than they had received.

Generally - with two exceptions - there was heavy criticism of the clinics: ‘they drag their feet’,’they tell us just go and sit there we will call you’. A repetitive refrain was: ‘We sit!’ This was the representation of the waiting experience which emerged in every discussion with both staff and mothers/caregivers (in both the individual interviews and the feedback meetings). The three QI priority areas that were identified at the mothers/caregivers’ meeting were logistics, attitude and communication (see Table 2).

**Table 2:** Mothers’priorities

Theme	Illustrative quotations
Logistics	‘’Honestly, it was hell being woken at 4 in the morning and waiting till the doctor came at 9”. ‘’We had to sleep on 2 blankets on the floor” ‘’I waited from 4 in the morning and the queue went round the clinic” ‘’It needs a play area for the kids”
Attitude	‘’They shouted me. They said it was my fault (that the child was so ill) and that I had no shame.” ‘’She was so naughty, Sister…, (laughing) always making jokes” ‘’It is not all of them, we mustn’t take the negative and put it on top of all the positive, about 10 % are like that … the rest really try hard.” ‘’They turned me away (when she came late from work) and told me I would have to make a plan - I said I made a plan … I am here now!” ‘’I was so angry I was just ready for the fighting…” ‘’They were really good to me”
Communication	‘’I tried to explain and he (doctor) didn’t listen … he said my question was too difficult” ‘’I can talk to her about all my problems and she will always listen” ‘’They didn’t tell me why she was swelling” ‘’I know I have to give her mince and maize and bananas”

The mothers/caregivers feedback meeting was attended by 5 mothers, 2 of their children and the core team members. Mothers had agreed that the hospital was the most central venue and the meeting was held there, with refreshments and toys for the children. The meeting took two hours, with the film being presented and then the emotional mapping exercise being done. Everyone was asked to write down their thoughts while the film was being played, which they did. In the emotional mapping exercise (see Figs. 4 and 5) the service area which appeared to raise the most concern was the children’s ward with words like ‘stressed’, ‘rude’, ‘disrespected’ and ‘powerless’ being used; this contrasted strongly with staff members mostly positive impression of the same ward. Other services like the OPD, dietician, social worker and X-rays were mostly perceived by mothers as positive experiences with words like ‘confident’, ‘hopeful’ and ‘valued’ being chosen to describe their experiences at these points.

Thereafter in the meeting, from these two exercises, a prioritised list was drawn up for discussion at the combined patient/staff meeting which included attitudes of staff, waiting time and communication.

### The joint feedback event

A third, combined feedback meeting with staff and mothers was held where the 40 min compilation film was presented, as well as the synopsis of the staff and mothers’ QI priorities. The core team together with 16 staff members and 5 mothers (with 3 children) attended this event. After discussion of all the issues which had already emerged, three mixed co-design groups were formed which brainstormed the most important combined priorities resulting in 3 thematic areas:

- attitudes and communication
- waiting
- practical issues.

Everyone was then allocated to a co-design team with at least one mother in each of the teams.

There was a vibrant experience of engagement in this meeting with a patient saying that the value of this process was that, ‘we will all understand each other’s feelings, behaviours, and attitudes, and another indicating, ‘I was very happy to be contributing in the meeting’. Staff, likewise reported it was helpful to hear ‘their concerns rather than thinking you know what they are’ and from a patient who complained that ‘doctors have very bad attitude’. Each individual attendee at the meeting articulated what they had experienced, contributing to a tapestry of ‘aesthetics’.

### Co-design teams

After 2 further meetings of each of the 3 co-design teams that were formed at the joint feed back meeting, a total of 38 concrete, practical QI interventions were suggested (see Table 3 which is based on a format devised by Locock et al. [26]). Of these, 25 were implemented in the research study timeframe (January to August 2015) and 5 others were being discussed or were temporarily put aside due to financial constraints eg reorganising the filing registry department to have a paediatric component and colour coding paediatric files.. The remainder were considered to be inappropriate for the time being due to lack of management support. These included moving the paediatric ward from the 3^rd^ floor to the ground floor in order for the children to have an outside play area and integrating the general paediatric outpatients department with the HIV children’s clinic.

**Table 3:** Interventions suggested and implemented by co-design teams [26]

Improvement activity: already implemented or planned for	Small scale	Process design within team	Process design between services	Process design between organisations
Small screen for privacy at reception	x			
Triage by senior staff at ED	x			
Lazyboys (reclining chairs) for long term mothers - donations sourced		x		
Mattresses for mothers in the ward	x			
All referred patients through clinic doctors			x	
Tracing of children through CHWs			x	
Tea for mothers in morning	x			
Debriefing of paediatrics staff by university psychology department				x
Schooling				x
Library				x
Organisation of child files at clinics			x	
Ill children straight to ward from ED without waiting for results (with SOP)	x			
Downreferral criteria in POPD	x			
Departmentalisation		x		
Prescription pathway at clinics- plan/SOP			x	
Ward orientation tool for admitted patients	x			
Soundproof area in POPD (moved to doctors room)	x			
Urns for Paediatric ward	x			
Psychological support for mothers				x
Play area at wellness		x		
Q marshals at clinics			x	
Q marshals at hospital reception	x			
Positive attitude workshops by subdistrict psychologist planned			x	
Time appointments at POPD		x		
Point of care blood test machine in ED – on priority equipment list		x		

A final celebration event was held in September 2015 to give feedback on progress - the achievements and difficulties encountered in the project - to the managers of the services, staff and mothers. This was attended by 16 people. The EBCD process includes the following: ‘’ ..Holding a celebratory event for everyone involved six to nine months after the joint patient–staff event is a simple but important way of thanking participants, reporting back on what has been achieved, and providing a clear ending point to this part of the project. This may also act as a catalyst for future projects” [28].

Based on this combined group’s input, a small task team including healthcare workers from primary care and the hospital was chosen to continue with the work and to keep the importance of remaining aware of patient experience alive in the ongoing wider QI work. Whilst EBCD became part of the still ongoing QI project – placing it within a stable, improvement milieu with some continuity - it is not possible to predict to what extent change will continue. The most recent malnutrition death rate at this hospital has been 0 over the last 3 months. One would hope that the QI project incorporating EBCD has made some contribution towards this,. A separate but related ongoing QI project on child antiretroviral therapy (ART) in primary care commenced in 2014, as most of the children from the hospital HIV clinic have been down referred to primary care clinics. This was initiated as a standard QIP but may be considered for an EBCD intervention, with the experience gained in the current research.

## Discussion

EBCD is a clearly defined process which allows patient perceptions and emotions to emerge as part of a planned change or improvement process. The approach also acknowledges the importance of staff contributions and - crucially - the important contribution that co-design (staff and patients working together as partners) can make to QI ‘work’. In longer term evaluations of the approach it has been reported that EBCD engendered positive working relationships whilst acknowledging issues like the locus of control and the challenge of implementing change processes in complex environments (including the discrepancy between patients’ expectations of the speed of improvement and the reality) [21].

Achievements in this particular EBCD project were the ability to explore authentic feelings and experiences of patients and staff regarding their exposure to the paediatric health care services in this subdistrict, the acknowledgement of a paternalistic system and the potential to change this by co-creating solutions, innovations with practical outcomes that were a synergy between previously disparate groups (providers and clients), a number of small sustained changes to improve the ‘’aesthetics” of the system and a measure of debriefing regarding staff and mothers/caregivers.

Emotions and motives for working in healthcare services were unexpected findings amongst the staff. It was interesting that passion characterised many of the staff stories as well as awareness of feelings of guilt and blame; often private family stories were shared as reasons for working with HIV patients or children. The level of engagement from staff in discussing improvement was similarly unpredicted and humbling. Whereas the health care workers contributed more to the pragmatic solutions, patients made more emotive judgments based on their personal experiences. Such experiences were both positive and negative and were captured both in the emotional mapping and also at all the feedback meetings. There were similarities in the themes from staff and patients and solutions were sought, often being generated in the collaborative discussions.

Challenges. As with the project described here, Bowen et al. – service designers who have worked with EBCD in an English hospital – have highlighted that conflict and/or disagreements between staff and patients cannot be completely removed [27]. The process of recruiting patients and retaining them also proved challenging. Studies using EBCD in Emergency Departments in Australia also found the recruitment and retention of patient participants to be difficult [13]. Language was a problem in some cases as English was not the first language for most of the staff and parents. The emotional value of the films is acknowledged but at the same this may act as a constraint for future projects; duplicating this method in the future would be time consuming and costly. It may be necessary to emulate the route followed in the United Kingdom, where previously filmed patient interviews could be used as ‘trigger’ films in improvement work [26, 31]. The lack of financial resources to implement some structural suggestions was acknowledged; these were not discarded entirely and were either ‘parked’ as future possibilities or donors were sought. In spite of the barriers, the attitude of co-operation from most of the hospital management was humbling, as was the enthusiasm of the patients that remained involved.

## Conclusion

In line with discourses of ‘customer/client care’ in other industries, the importance of engaging patients to improve the quality of the healthcare services provided to them has proven to be valuable and productive. In this research project, the integration of mothers/caregivers and staff into co-design teams has led to concrete practical improvements in a paediatric service, and also spanning the boundaries of hospital and primary health clinic care.

As QI practices have not yet become routine in healthcare organisations in South Africa, staff members were almost as much of an untapped source of ideas and perceptions as patients. The energy generated between the two groups, based largely on feelings about being ‘together’ as part of the same service, lead to 38 possible solutions, all based on discomfort or negative feelings about certain aspects of care.

## Abbreviations

ART, Antiretroviral therapy; BPN, Bronchopneumonia; EMTCT, Eradication of Mother to child transmission; GE, Gastroenteritis; OPD, Outpatients department; PHC, Primary Health Care; QI, Quality Improvement; UNICEF: United Nations Children’s Fund; WHO, World Health Organisation.

## Additional file

Additional file 1: Interviews from EBCD toolkit. (DOCX 16 kb)
