# Supplementary material for: Improving childhood nutrition and wellness in South Africa: involving mothers/caregivers of malnourished or HIV positive children and health care workers as co-designers to enhance a local quality improvement intervention
Source: BMC Health Serv Res. 2016 Aug 5;16:358. doi: 10.1186/s12913-016-1574-4 (PMC4975875; doi:10.1186/s12913-016-1574-4)
Supplement: Additional file 1: — Interviews from EBCD toolkit. (DOCX 16 kb) [file 12913_2016_1574_MOESM1_ESM.docx]

Interviews (from EBCD Toolkit)

**Script and interview schedule for staff interviews**

Thank you for agreeing to take part in this project. The aim is to improve the experiences of both those providing and receiving cancer services within our hospitals. The project is part of a wider programme of work, the goal of which is to enhance the way in which care is delivered. We are using an approach called experience-based co-design (EBCD). This approach provides a unique opportunity for staff and patients to work together in redesigning services in order to improve patient and staff experiences. Today, we wish to understand your experience of delivering the service to patients in order to develop services. With your consent, we would like to tape record the interview but the data gained from the interview will be made anonymous and you will also have the opportunity to review your interview transcript if you wish.

**1 Introduction**

So let us start with your background and how you came into your role.

• Tell me what your role is

• Which hospital/trust do your patients come from?

**2 Staff experiences**

• Can you tell me what is it like working in this service?

• What’s good about working here?

• Can you give me an example of a positive experience you have had?

• What’s not so good about working here?

• Can you give me an example of a negative experience?

• Can you describe a typical day’s work in this service?

• What do you think are the main problems with this service from the point of view of staff?

• How does working in this service compare to other places you have worked or are working?

• (*For consultants and clinical nursing specialists working in more than one hospital*) What are the differences between the two hospitals?

• (*If staff talk about politics within the service*) How does this issue impact on the patient experience?

*[Interviewer to summarise list of positive aspects and problems]*2

**3. Perceptions of patient experience**

• What do you think it is like being a patient in this service?

• What are your perceptions of the service you are providing to patients?

• Which patient needs are met? Not met?

• What do you think are the major problems faced by patients?

• What could be improved for patients in this service?

• (*For staff seeing patients from more than one hospital*) Do you notice differences in the experiences of patients coming from different hospitals?

• In your opinion, what are the major ‘touch points’ or critical moments in the patient journey (the things or events that really shaped their overall experience)?

• Overall do you think that you provide the care you would like for yourself and your family?

• Would you be happy if a member of your family was going to be treated here?

• What aspects of the service would you be happy or unhappy with?

**4. Improving the service**

• What do you see are the main priorities for improving the service from the staff point of view?

• How should things develop?

• What other things do you feel would help to improve your experience and the experience of other staff in this service?

• What do you think patients would identify as things that would help to improve patient experiences?

• In your opinion, where might we begin to improve the patient experience around this service?

*[Interviewer to summarise list of priorities]*

**THANK YOU**
